# Risk Factors of Ischemia Reperfusion Injury After PCI in Patients with Acute ST-Segment Elevation Myocardial Infarction and its Influence on Prognosis

**DOI:** 10.3389/fsurg.2022.891047

**Published:** 2022-06-07

**Authors:** Li Zhang, Lingqing Wang, Luyuan Tao, Changgong Chen, Shijia Ren, Youyou Zhang

**Affiliations:** ^1^ Department of Cardiovascular Medicine, The First People’s Hospital of Taizhou City, Taizhou, China; ^2^ Department of Endocrinology, The First People’s Hospital of Taizhou City, Taizhou, China

**Keywords:** ST-segment elevation myocardial infarction, percutaneous coronary intervention, ischemia reperfusion injury, risk factors, prognosis

## Abstract

**Purpose:**

To explore the risk factors of ischemia reperfusion injury (IRI) after percutaneous coronary intervention (PCI) in patients with acute ST-segment elevation myocardial infarction (STEMI) and its influence on prognosis.

**Methods:**

The clinical data of 80 patients with STMEI undergoing PCI in our hospital from June 2020 to June 2021 were collected. According to whether IRI occurred after PCI, STMEI patients were divided into IRI group and non-IRI group. The basic information, clinical characteristics, examination parameters and other data of all patients were collected, and the prognosis of the two groups was observed. Risk factors were analyzed by fitting binary Logistic regression model. The survival prognosis was analyzed by Kaplan-Meier survival curve.

**Results:**

Logistic regression analysis showed that type 2 diabetes mellitus (T2DM), pre-hospital delay time (PHD) and door-to-balloon expansion time (DTB) were the influencing factors of IRI in patients with STMEI (*p *< 0.05). MACE occurred in 11 cases (32.35%) in the IRI group and 13 cases (28.26%) in the non-IRI group. Log-rank test showed *p *= 0.503, indicating no statistically significant difference.

**Conclusion:**

T2DM, PHD and DTB were the influencing factors of IRI in patients with STMEI, and IRI will not reduce the prognosis of patients.

## Introduction

ST-segment elevation myocardial infarction (STEMI) is mainly caused by unstable plaque detachment in coronary artery to form thrombus, which leads to acute myocardial ischemia and necrosis. The main clinical symptoms are persistent ischemic chest pain, elevated serum myocardial injury markers and elevation of ST segment of electrocardiogram (1). STEMI has an acute onset and rapid onset. Patients may suffer from myocardial injury and necrosis in a short period of time, even death in severe cases, which poses a serious threat to the life and health of patients (2). With the continuous development of medical technology, the early mortality of STEMI in clinic has dropped significantly, from 13% in 1986 to <4% in 2000, which is mainly due to the application of early opening of infarction-related vascular technology (3). Percutaneous coronary intervention (PCI) is the most common method for the treatment of STEMI. It can recanalize infarct-related arteries, restore myocardial perfusion and restore blood supply, and has been widely carried out in China (4). However, with the deepening of relevant research, scholars have found that effective reperfusion therapy may not only restore the forward blood flow of infarct-related arteries, but also lead to the further aggravation of myocardial ischemic injury, that is, ischemia reperfusion injury (IRI) (5). IRI can be manifested as severe slow arrhythmia, malignant ventricular arrhythmia, cardiac insufficiency and sudden drop of blood pressure after vascular opening, resulting in acute and chronic organ failure, even sudden death, which affects postoperative recovery, so it has been widely valued by doctors in recent years (6). Therefore, it is particularly important to identify the risk factors of IRI in STEMI patients as early as possible clinically. We aim to observe the influencing factors of IRI in STEMI patients after PCI, and whether the occurrence of IRI affects the prognosis of patients.

## Materials and Methods

### Research Object

The clinical data of 80 patients with STMEI undergoing PCI in our hospital from June 2020 to June 2021 were collected. According to whether IRI occurred after PCI, STMEI patients were divided into two groups: 34 cases in IRI group and 46 cases in non-IRI group. Inclusion criteria: Patients meet the diagnostic criteria of STMEI (7); The duration of onset of chest pain ≥30 min; The patient can accurately tell the doctor the specific time of onset; Complete clinical data. Exclusion criteria: those who failed to receive PCI treatment within 12 h of onset due to various reasons; Coronary angiography confirmed incomplete occlusion of criminals’ blood vessels; Patients who died during PCI or 24 h after PCI; Patients with active visceral hemorrhage, cardiogenic shock; Liver and renal insufficiency; Combined with malignant tumor.

The diagnostic criteria of IRI were as follows: ① Severe bradycardia, hypotension and frequent premature ventricular contractions within minutes after opening coronary artery vessels during interventional therapy; ② Serious ventricular arrhythmias still occur after drug therapy and/or electrocardioversion and electrodefibrillation; ③ coronary angiography detected coronary angiography TIMI ≤ grade 2, combined with thrombus, dissection or spasm.

### Research Methods

All patients received comprehensive treatment measures before operation such as oxygen inhalation, sedation, analgesia, ECG monitoring. The patients were treated with nitrates, *β*-blockers, heparin and angiotensin converting enzyme inhibitors. Within 12 h of onset, PCI was completed under the guidance of angiography system. Routine percutaneous puncture of the radial artery or femoral artery, coronary angiography was performed, and after the coronary artery disease was identified, the location of the infarction-related artery disease was determined, stent placement was performed, and PCI treatment was completed immediately. PCI was performed by experienced physicians. All patients were treated with double antiplatelet aggregation therapy after operation. If there were no contraindications, nitrates, *β* -blockers and other drugs were added.

The clinical data of all patients were collected, including: ① age, sex, hypertension, type 2 diabetes mellitus (T2DM), hyperlipidemia, smoking history (>20 cigarettes/day) and alcoholism history (>150 mg/day); ② Admission systolic blood pressure, admission diastolic blood pressure, admission heart rate, pre-hospital delay time (PHD) and door-to-balloon expansion time (DTB); ③ White blood cell count (WBC), platelet count (PLT), total cholesterol (TC) and low density lipoprotein cholesterol (LDL-C) at admission.

Telephone and outpatient follow-up were conducted 6 months after operation to record the prognosis of patients, including major adverse cardiovascular events (MACE) such as readmission, recurrent myocardial infarction, revascularization, fatal arrhythmia, heart failure, stroke, bleeding of vital organs and cardiogenic death. Patients who died or lost follow-up during the follow-up period were excluded.

### Statistical Methods

Used SPSS 22.0 software to process. The measurement data was expressed by mean ± standard deviation, and the comparison was made by *t* test. The count data are expressed by ratio, and the comparison is made by *χ*^2^ test. Risk factors were analyzed by fitting binary Logistic regression model. The survival prognosis was analyzed by Kaplan-Meier survival curve, and the comparison was made by Log-rank test. Inspection level *α* = 0.05.

## Results

### Basic Information of Patients

There were significant differences in hypertension and T2DM between IRI group and non-IRI group (*p *< 0.05) (Table 1).

**Table 1:** Basic information of patients (*n*,%).

Items	non-IRI group (*n* = 46)	IRI group (*n* = 34)	*χ*^2^ value	*p-*value
Age (years)			1.507	0.220
≤60	28 (60.87%)	16 (47.06%)		
>60	18 (39.13%)	18 (52.94%)		
Sex			0.101	0.751
Male	30 (65.22%)	21 (61.76%)		
Female	16 (34.78%)	13 (38.24%)		
Hypertension			3.942	0.047
With	13 (28.26%)	17 (50.00%)		
Without	33 (71.74%)	17 (50.00%)		
T2DM			4.557	0.033
With	10 (21.74%)	15 (44.12%)		
Without	36 (78.26%)	19 (55.88%)		
Hyperlipoidemia			0.001	0.969
With	12 (26.09%)	9 (26.47%)		
Without	34 (73.91%)	25 (73.53%)		
Smoking history			0.077	0.782
With	27 (58.70%)	21 (61.76%)		
Without	19 (41.30%)	13 (38.24%)		
Alcoholism history			0.621	0.431
With	15 (32.61%)	14 (41.18%)		
Without	31 (67.39%)	20 (58.82%)		

### Clinical Characteristics of Patients

There were significant differences in PHD and DTB between IRI group and non-IRI group (*p *< 0.05) (Table 2).

**Table 2:** Clinical characteristics of patients (*n*, $\bar{x}\pm s$, %).

Items	non-IRI group (*n* = 46)	IRI group (*n* = 34)	*t/χ*^2^ value	*p* value
Admission systolic blood pressure (mmHg)	139.64 ± 14.29	135.64 ± 13.88	1.399	0.165
Admission diastolic blood pressure (mmHg)	78.92 ± 10.34	76.50 ± 10.16	1.042	0.300
Admission heart rate (times /min)	83.84 ± 11.39	87.73 ± 10.60	1.554	0.124
PHD (h)			7.020	0.008
≤6	30 (65.22%)	12 (35.29%)		
>6	16 (34.78%)	22 (64.71%)		
DTB(min)			9.934	0.007
<90	16 (34.78%)	2 (5.88%)		
90–180	24 (51.17%)	23 (67.65%)		
>180	6 (13.04%)	9 (26.47%)		

### Examination Parameters of Patients

There was no significant difference in WBC, PLT, TC and LDL-C between IRI group and non-IRI group (p > 0.05) (Table 3).

**Table 3:** Examination parameters of patients (*n*, $\bar{x}\pm s$).

Items	non-IRI group (*n* = 46)	IRI group (*n* = 34)	*t* value	*p* value
WBC(×10^12^/L)	11.35 ± 1.54	11.08 ± 1.69	0.743	0.459
PLT(×10^9^/L)	201.66 ± 30.74	208.81 ± 28.57	1.059	0.293
TC(mmol/L)	4.75 ± 0.72	4.98 ± 0.76	1.379	0.171
LDL-C(mmol/L)	2.71 ± 0.50	2.92 ± 0.63	1.661	0.100

### Multivariate Analysis of IRI in Patients with STMEI

Logistic regression analysis showed that T2DM, PHD and DTB were the influencing factors of IRI in patients with STMEI (p < 0.05) (Tables 4, 5).

**Table 4:** Multi-factor assignment.

Independent variable	Assignment
Hypertension	With = 1, Without = 2
T2DM	With = 1, Without = 2
PHD	≤6 h = 1, >6 h = 2
DTB	<90 min = 1, 90–180 min = 2, >180 min = 3

**Table 5:** Multivariate analysis of IRI in patients with STMEI.

Variable	*B* value	*SE* value	*Walds* value	*OR* value	*95%CI*	*p-*value
Hypertension	0.468	0.242	3.739	1.596	0.993–2.566	0.872
T2DM	0.339	0.157	4.662	1.403	1.031–1.909	0.038
PHD	0.591	0.185	10.205	1.806	1.256–2.595	0.031
DTB	0.654	0.163	16.098	1.923	1.397–2.647	0.015

### Follow-up of Patients

**Figure 1:**
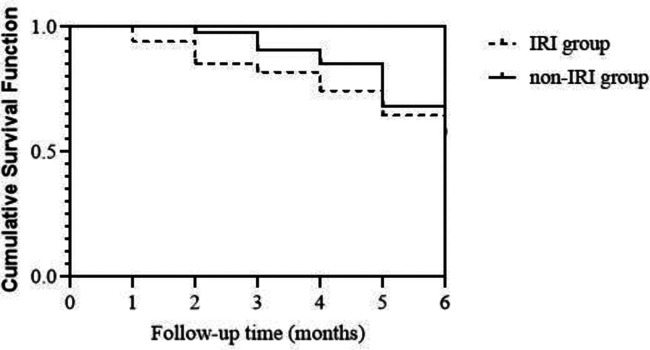
Follow-up of patients.

As of January 2022, all patients were followed up. MACE occurred in 11 cases (32.35%) in the IRI group and 13 cases (28.26%) in the non-IRI group. Log-rank test showed *p *= 0.503, indicating no statistically significant difference (Figure 1).

## Discussion

STEMI can cause arrhythmia, myocardial injury and other symptoms, and severe conditions can endanger the patient’s life. PCI can open infarct-related vessels and restore distal myocardial perfusion, which is an efficient and safe treatment for STEMI (8). PCI for STEMI patients can restore the effective blood supply of coronary artery as soon as possible. However, reperfusion therapy after ischemia may further cause myocardial injury, which can seriously affect the surgical efficacy and prognosis. The main mechanisms of IRI are oxidative stress, intracellular calcium overload, destruction of microvascular structure and function, and opening of mitochondrial membrane permeability transformation pores (9, 10). Therefore, exploring the related factors of IRI has becomes an important issue to prevent the occurrence of IRI in STEMI patients after PCI.

This study found that T2DM is the influencing factor of IRI in patients with STMEI. Literature reports at home and abroad show that there are inconsistent research results on the issue of DM on IRI. Dia’s team research showed that patients without DM often fail to establish a good collateral circulation before acute occlusion due to their previous health and lack of long-term chronic myocardial ischemia preconditioning before acute occlusion of criminals’ blood vessels. Therefore, compared with STEMI patients with DM, patients without DM have stronger reaction to acute ischemic injury and are more prone to IRI (11). Muráriková’ s team believed that because the heart of DM patients may adapt to the changes caused by DM and increase tolerance to ischemic injury, the recovery of DM patients after IRI is better than that of patients without DM, with fewer cardiovascular events (12). However, there are also different research results on the relationship between DM and IRI in clinic. Badalzadeh’s team found that STEMI patients with DM have a higher risk of IRI, which may be due to the combined effects of changes of glucose and lipid energy metabolism based on insulin resistance, enhanced oxidative stress and systemic inflammatory responses and ion channel dysfunction (13). Some scholars also believe that long-term chronic hyperglycemia can affect the function and structure of platelets, damage vascular endothelial cells, cause microvascular lesions, and weaken the repair ability of vascular endothelial cells, leading to coronary circulation disorder. DM is one of the main causes of IRI (14). In addition, it has been reported that T2DM has a series of metabolic syndromes caused by insulin resistance, while T1DM does not have insulin resistance in the early stage, and with the prolongation of the disease course, T1DM also gradually develops insulin resistance. Clinically, DM patients with STEMI undergoing PCI often have a long course of disease, and almost all of them have insulin resistance, so the IRI is seriously damaged (15, 16).

In this study, under the condition of PHD ≤ 6 h and DTB < 90 min, the probability of IRI in patients with STMEI is small, while when PHD exceeds 6 h and DTB exceeds 180 min, the probability of IRI is significantly increased. Guidelines have shown that opening occluded blood vessels within 12 h of onset of STMEI patients can have beneficial effects on the patient (17). There is a time window from acute coronary artery occlusion to transmural myocardial necrosis, which is about 6 h. Recanalization of coronary arteries within this time window can save ischemic myocardium on the verge of necrosis, promote the body to recover the forward blood flow and occlude blood vessels as soon as possible, and improve the myocardial microcirculation perfusion level (18, 19). The delay in the visit of patients with STMEI can seriously affect the follow-up cardiac function, and early seeing a doctor can provide patients with the opportunity to obtain a good prognosis. In addition, the earlier the treatment of the first balloon dilatation is performed, the greater the benefit of patients. Bruce’s team conducted a long-term follow-up of patients with acute myocardial infarction who underwent PCI. The results found that prolongation of DTB could increase the risk of in-hospital death and late death, and DTB > 2 h was an independent risk factor of death (20). De Luca’s team showed that with the delay of DTB, the one-year mortality rate of patients with STMEI increased, and the relative risk of death was 1.08 for every 30 min increase in DTB (21, 22). Scholars at home and abroad believe that DTB < 90 min is the best time for the first balloon dilatation, and the shortest DTB can greatly reduce the incidence of short-term and long-term adverse cardiac events after PCI (23, 24). Prehospital delays mainly include patient delays and transport delays. The patient delay is due to the patient’s lack of awareness of the visit to the clinic, which leads to the extension of PHD. Transport delay is due to the large number of patients in the hospital, which leads to a delay in the time of transfer and treatment. In clinical practice, out-of-hospital ECG can greatly shorten PHD and DTB, which is beneficial to quickly determine whether IRI occurs in STMEI patients, so as to make correct treatment decisions. At the same time, an in-hospital emergency service system composed of well-trained ambulance teams is also crucial. The development of the in-hospital emergency service system can avoid the transition of wards or coronary heart disease care units, thereby shortening the time for transferring patients in the hospital and further reducing DTB. In addition, medical staff need to strengthen the people’s awareness of STEMI, and identify and treat IRI in patients with STMEI as soon as possible.

In addition, we also found that the occurrence of IRI has no significant influence on whether there is MACE in patients with STMEI. The results suggest that although IRI may have some adverse effects on patients with STMEI, it is a pathophysiological change in a short time during myocardial reperfusion, and IRI in STMEI patients after PCI will not reduce the prognosis of patients. Medical staff need to improve the ability to identify the risk of IRI, and to carry out risk prevention and effective treatment for high-risk factors, without changing the treatment strategy due to IRI.

## Conclusion

To sum up, T2DM, PHD and DTB were the influencing factors of IRI in patients with STMEI, and IRI will not reduce the prognosis of patients. This study is a single-center, small-sample study, and there is a lack of longer follow-up for patients. We need to improve the research scheme in the future.

## Data Availability

The original contributions presented in the study are included in the article/Supplementary Material, further inquiries can be directed to the corresponding author/s.
